# An unaddicted impact factor

**DOI:** 10.1186/1129-2377-14-58

**Published:** 2013-07-04

**Authors:** Paolo Martelletti

**Affiliations:** 1 Department of Clinical and Molecular Medicine, Sapienza University, Rome, Italy

## Making the natural evolution

There is no need of pageantry to celebrate this ultimate success of The Journal of Headache and Pain (TJHP), reporting an increase in Impact Factor (IF) values, rising up to 2.779 for 2012, with an increase of 14,5% versus its own 2011 IF and a superb 35,5% versus 2010 IF.

This growth is physiologically sane, rooted on citations widely distributed on the majority of the published papers. We did not benefit from citation peaks given to innovative RCTs or inescapable classifications.

Beyond doubts in few years we turned an ailing journal into today’s global cultural platform [1]. “We” means all of us. The scientists who ever since accepted to share a new project, the opponents who gave us sense of direction, the Publisher which strongly supported every innovative idea, the two affiliated Societies, Lifting The Burden and European Headache Federation which concretely offered TJHP their best scientific products [2,3], the authors who chose either to publish here or to cite TJHP elsewhere. I am talking about all the authors, the ones who publish on journals with top or low IF, the researchers who follow a conspicuous growth of their own Hirsch index and the physicians who privilege the clinical aspects. A citation is always worth one unit, both in top class and the economic one. Today these are the ongoing rules.

The authors who chose to publish here have gained a complete absence of political varnish on editorial decisions, a pit-stop speed on the various phases of publication process and, as an added value and only in case of missed publication for low priority, their immediate resubmission towards other open access related journals without loosing the peer-reviewing phases already taken. This is the SpringerOpen standard.

A particular warm thank goes to the many reviewers who daily accept revisions with particularly strict deadlines. They are the spine of TJHP. Thanks to them, in less than two months the editorial process is usually complete, from the submission to the eventual appearance on PubMed. Many have appreciated this.

## A well-timed open access choice

Still many confuse open access and Article Processing Charge (APC). TJHP is open access since 2011 and has introduced the APC since 2013. Neither of these two factors has had a preponderant impact on TJHP evolution. The courage to precociously undertake the difficult open access choice, strategy today widely accepted and much used, has allowed a sowing of TJHP contents. APC, from many considered as an obstacle in the beginning, has not been lived as such in the end. APC guarantees the completeness in the budgeting process of a structured research project. Many of us remember that, more than some years ago, the budget of a research project must include an economic coverage for reprints. What was it if not an *ante litteram* APC?

Moreover, submission flow has not been affected from this new procedure. It has rather produced a useful waste of low-quality remains resulting in an evident fall of immediate rejection for unsound submissions.

## Expectations tempered with caution

The cultural liberalism promoted by TJHP in headache research, connected with its strong educational vocation, allowed new research groups to appear in the headache disorders area. TJHP has always had as cornerstone the removal of the barriers which impoverish both basic and clinical research, highlighting the necessity to open this parterre to many more actors in order to reach effective results, being evident the imbalance between the headache disorders epidemiological vastness and the correlated scientific production.

It is however essential to notice that, from an accurate analysis of the headache disorders editorial scenario, all area journals were rewarded and this witnesses that there is still room to further grow. The new frontiers strategy, in terms of sharing borderline headache disorders with other disciplines than those usual ones, must be favored and encouraged with *ad hoc* partnerships. This might be rewarding for everybody, scientists and clinical experts, readers and patients.

The race “we” have in the offing is not over.
